# The Effect of Laparoscopic Gastric Bypass Surgery on Insulin Resistance and Glycosylated Hemoglobin A1c: a 2-Year Follow-up Study

**DOI:** 10.1007/s11695-020-04599-w

**Published:** 2020-04-20

**Authors:** Erik Stenberg, Eva Rask, Eva Szabo, Ingmar Näslund, Johan Ottosson

**Affiliations:** 1 Department of Surgery, Faculty of Medicine and Health, Örebro University, Örebro, Sweden; 2 Department of Surgery, Örebro University Hospital, SE-70185 Örebro, Sweden; 3 Department of Endocrinology, Faculty of Medicine and Health, Örebro University, Örebro, Sweden

**Keywords:** Insulin, Insulin resistance, Gastric bypass, Bariatric surgery, Postoperative outcome

## Abstract

**Background:**

Bariatric surgery improves insulin sensitivity and secretion in patients with type 2 diabetes, but the effect on patients with prediabetes or even normal glucose tolerance deserves further consideration.

**Methods:**

Cohort study including patients operated with laparoscopic Roux-en-Y gastric bypass surgery (LRYGB) between November 2012 and June 2017 at the Örebro University Hospital (*n* = 813) with follow-up of 742 patients 2 years after surgery. Fasting insulin, glucose, glycosylated hemoglobin (HbA1c), and homeostatic model assessment of insulin resistance (HOMA-IR) were analyzed at baseline and 2 years after surgery for patients with overt type 2 diabetes, prediabetes, or non-diabetes.

**Results:**

Fasting insulin levels improved for all groups (diabetics baseline 25.5 mIU/L, IQR 17.5–38.0, 2 years 7.6 mIU/L, IQR 5.4–11.1, *p* < 0.001; prediabetics baseline 25.0 mIU/L, IQR 17.5–35.0, 2 years 6.7mIU/L, IQR 5.3–8.8, *p* < 0.001; non-diabetics baseline 20.0 mIU/L, IQR 14.0–30.0, 2 years 6.4 mIU/L, IQR 5.0–8.5, *p* < 0.001). HbA1c improved in all groups (diabetics baseline 56 mmol/mol, IQR 49–74 [7.3%, IQP 6.6–8.9], 2 years 38 mmol/mol, IQR 36–47 [5.6%, IQR 5.4–6.4], *p* < 0.001; prediabetics baseline 40 mmol/mol, IQR 39–42 [5.8%, IQR5.7–6.0], 2 years 36 mmol/mol, IQR 34–38 [5.5%, IQR 5.3–5.6], *p* < 0.001; non-diabetics baseline 35 mmol/mol, IQR 33–37 [5.4%, IQR 5.2–5.5]; 2 years 34 mmol/mol, IQR 31–36 [5.3%, IQR 5.0–5.4], *p* < 0.001). HOMA-IR improved in all groups (diabetics baseline 9.3 mmol/mol, IQR 5.4–12.9, 2 years 1.9 mmol/mol, IQR 1.4–2.7, *p* < 0.001; prediabetics baseline 7.0 mmol/mol, IQR 4.3–9.9, 2 years 1.6 mmol/mol, IQR 1.2–2.1, *p* < 0.001; non-diabetics 4.9 mmol/mol, IQR 3.4–7.3, 2 years 1.4 mmol/mol, IQR 1.1–1.9, *p* < 0.001).

**Conclusion:**

Insulin homeostasis and glucometabolic control improve in all patients after LRYGB, not only in diabetics but also in prediabetics and non-diabetic obese patients, and this improvement is sustained 2 years after surgery.

## Introduction

Diabetes and prediabetes are common conditions among patients with morbid obesity being considered for bariatric surgery [1–3]. Patients with diabetes carry a significant risk of developing micro- and macrovascular complications [4]. The risk of complications, both in type 2 diabetes and type 1 diabetes, is strongly correlated to the duration of the disease and to the level of hyperglycemia [5–7].The sooner the hyperglycemic state is corrected, the better the prognosis, and ideally, if the disease could be prevented, this would be the largest gain for the patient and the health-care system. Laparoscopic bariatric surgery has a low complication rate [2, 8], and is considered an effective treatment for diabetes in patients with morbid obesity [9–12], with documented reduction in micro- and macrovascular complications [13, 14]. Gastric bypass and sleeve gastrectomy have both been reported to reduce peripheral insulin resistance and improve glucose homeostasis in patients with type 2 diabetes and to some extent in patients without type-2 diabetes at the time of surgery [12, 15–23]. Previous studies in patients with impaired glucose tolerance or impaired fasting glucose are more scarce.

The aim of the present study was to evaluate the effect of gastric bypass surgery on glucose homeostasis and insulin resistance in patients with morbid obesity and overt diabetes mellitus, prediabetes, and normal glucose levels, respectively.

## Material and Methods

All patients operated with laparoscopic Roux-en-Y gastric bypass surgery (LRYGB) at the Örebro University Hospital in Sweden were eligible for inclusion in the study. Collection of samples for fasting serum insulin began at this institution in November 2012. In order to enable a 2-year follow-up, the inclusion period was defined as November 1, 2012, until June 30, 2017. Patients with missing data on fasting serum insulin at baseline and patients with type 1 diabetes were excluded from the study.

All patients were seen at baseline, day 30 and at 1 and 2 years after their operation. Some patients were unable to attend a follow-up visit at the hospital department and instead were followed up by telephone or mail contact. Fasting blood samples for insulin, glucose, and HbA1c were taken at baseline (prior to preoperative weight reduction) and at 1 and 2 years after surgery. Any postoperative changes to medical treatment for diabetes were made by the physician handling the diabetes.

All patients were kept on a low-calorie diet 4 to 6 weeks before surgery. The surgical procedures were all antecolic, antegastric LRYGB with a 50-cm long biliopancreatic limb and a 100-cm alimentary limb as described by Olbers and Lönroth [24]. One linear stapler was used to construct the gastroenterostomy, and the remaining defect was closed with a running absorbable suture. All mesenteric defects were closed to reduce the risk for internal hernia [25]. Postoperatively, oral fluids were started on the day of surgery.

Based on the recommendations of the American Diabetes Association (ADA), patients were divided into three groups: patients with overt diabetes, prediabetes, or non-diabetes. Prediabetes was defined as an HbA1c of 5.7–6.5% (39–48 mmol/mol) without medical treatment [26]. Diabetes was defined as ongoing medical treatment for diabetes or an HbA1c > 6.5% (> 48 mmol/mol) with or without medical treatment at baseline [26].

Change in weight was estimated as percentage total weight loss (%TWL = 100 x [initial weight – weight at 2-year follow-up] / initial weight) and excess BMI loss (%EBMIL = 100 x [initial BMI – BMI at 2-year follow-up] / [Initial BMI – 25]) comparing the change in weight between the weight prior and preoperative weight reduction with that 2 years after surgery.

### Outcome Measures

The primary outcome was insulin resistance (estimated by fasting serum insulin and HOMA-IR) 2 years after surgery. Secondary outcomes were HbA1c and fasting plasma glucose 2 years after surgery. HbA1c values were reported according to the reference standard of the International Federation for Clinical Chemistry and Laboratory Medicine (IFCC) and converted to the standards of the Diabetic Control and Complication Trial using a validated conversion table [27]. The homeostasis model assessment of insulin resistance (HOMA-IR) was based on the following equation: HOMA-IR = Glucose (mmol/L) x Insulin (mIU/L) /22.5 [28]. Patients receiving insulin treatment at baseline were excluded from the analyses of serum insulin and HOMA-IR.

Complete remission of diabetes was defined as HbA1c 6.0% (42 mmol/mol) without medical treatment, and partial remission was defined as HbA1c 6.0–6.5% (42–48 mmol/mol) without medical treatment in accordance with the recommendations of the American Society for Metabolic and Bariatric Surgery [29]. Controlled diabetes at follow-up was defined as an HbA1c < 6.5% (< 48 mmol/mol) with medical treatment.

### Statistical Methods

The Shapiro-Wilks test was used to test normal distribution. Due to the fact that no outcome had a normal distribution, the Wilcoxon signed-rank test was used to compare changes over time. Categorical parameters were analyzed with the Chi-squared test. Correlation between %TWL and improvement in insulin resistance over 2 years was estimated with the Spearman correlation test. Missing data were excluded from analyses. All calculations were made using SPSS version22 (IBM corporation, Armonk, NY, USA).

### Ethics

The study was approved by the Regional Ethics Committee in Uppsala, Sweden, and was conducted in accordance with the 1964 Helsinki Declaration and its later amendments.

## Results

During the inclusion period, 821 patients underwent primary LRYGB. After exclusion of eight patients with type 1 diabetes, 813 patients remained in the study. Follow-up was registered for 768 patients (94.5%) after 1 year and 742 patients (91.3%) after 2 years. Fasting serum insulin was available at baseline and at 2 years for 599 patients (73.7%), for HbA1c for 678 patients (83.4%), and for fasting plasma glucose for 675 patients (83.0%).

### Baseline Characteristics Are Presented in Table 1

Among the 129 patients with diabetes mellitus type 2 (according to the ADA definition [26]), 54 received oral medical treatment, 10 received insulin treatment alone, 30 received a combination of oral treatment and insulin, and 35 did not receive any medication for their diabetes.

**Table 1:** Baseline characteristics for patients with 2-year follow-up

Base-line characteristic	Mean ± SD, or n (%)
BMI, mean ± SD, kg/m^2^	42.1 ± 5.22
Age, mean ± SD, yrs	40.2 ± 12.03
Sex	
Female, n (%)	571 (77.0%)
Male, *n* (%)	171 (23.0%)
Comorbidity, *n* (%)	379 (51.1%)
Sleep apnea, *n* (%)	80 (10.8%)
Hypertension, *n* (%)	181 (24.4%)
Diabetes, *n* (%)	121 (16.3%)
Prediabetes, *n* (%)	177 (23.9%)
Dyslipidemia, *n* (%)	58 (7.8%)
Dyspepsia/GERD, *n* (%)	31 (4.2%)
Depression, *n* (%)	67 (9.0%)
Previous DVT/VTE, *n* (%)	18 (2.4%)

There were no missing data

Three procedures (0.4%) were converted to open surgery. Mean operation time was 84 ± 25.0 min. A postoperative complication occurred within 30 days in 56 patients (6.9%). One patient died during the first 90 days (0.1%). The most common early postoperative complication was abdominal pain (*n* = 15, 1.8%), followed by bleeding (*n* = 14, 1.7%), bowel obstruction/paralysis (*n* = 14, 1.7%), wound infection (*n* = 8, 1.0%), leakage (*n* = 6, 0.7%), nutritional deficiency (*n* = 3, 0.4%), abscess/deep abdominal infection (*n* = 3, 0.4%), and pulmonary complication (*n* = 3, 0.4%).

Patients had lost on average 39.8 ± 13.1 kg 2 years after surgery, and %EBMIL 2 years after surgery was 84.8 ± 23.7% and %TWL 33.1 ± 8.5%.

At baseline, 186 patients were classified as having prediabetes. At the 2-year follow-up, of the 164 patients with information available on diabetes status, including HbA1c, 130 were no longer classified as having prediabetes (79.3%). One patient with prediabetes, hypertension, and dyslipidemia at baseline had an HbA1c level of 6.9% (52 mmol/mol) at follow-up and seemed thus to have developed diabetes. None of the 399 non-diabetic patients with available information on diabetes status and HbA1c at the 2-years follow-up developed diabetes during the follow-up time.

At the 2-year follow-up, of the 129 patients with type 2 diabetes at baseline, 118 had information available on their diabetes status including HbA1c. Of these, 64 (54.2%) had complete remission of their diabetes without medical treatment, 8 (6.8%) had partial remission without medical treatment, 18 (15.3%) had controlled diabetes, and 28 (23.7%) had HbA1c ≥ 48 mmol/mol despite medical treatment.

**Fig. 1:**
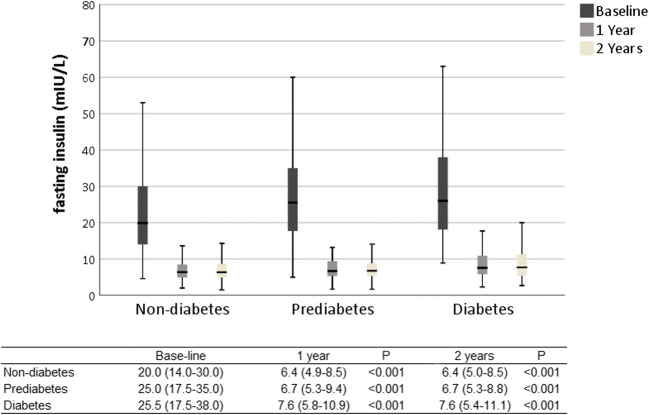
Clustered boxplot of mean fasting serum insulin at baseline, 1 and 2 years after surgery with interquartile range

**Fig. 2:**
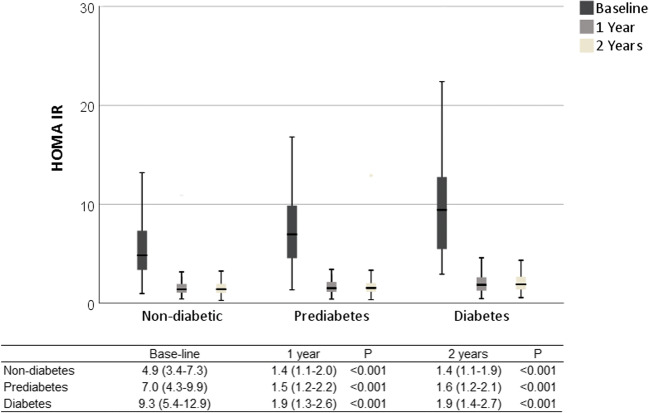
Clustered boxplot of mean HOMA-IR at baseline, 1 and 2 years after surgery with interquartile range

**Fig. 3:**
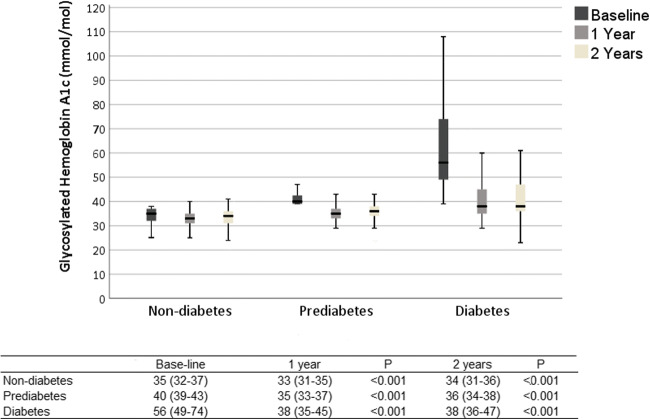
Clustered boxplot of mean glycosylated hemoglobin A1c at baseline, 1 and 2 years after surgery with interquartile range

Insulin, HbA1c, and HOMA-IR levels improved in all groups after surgery (Figs. 1, 2, and 3). Fasting plasma glucose levels (mmol/L) also improved in the non-diabetic group (baseline 5.5, IQR 5.2–5.9, 2 years after surgery 5.0, IQR 4.8–5.2), prediabetics (baseline 6.0, IQR 5.6–6.6, 2 years after surgery 5.2, IQR 5.0–5.5), and diabetics (baseline 8.5, IQR 7.1–10.3, 2 years after surgery 5.7, IQR 5.2–6.8; *p* < 0.001 for all comparisons).

Percentage total weight loss was correlated to improvement in insulin levels in patients of all subgroups, without diabetes (Spearman rank correlation coefficient, *r* = − 0.213, *p* < 0.001), prediabetics (*r* = − 0.424, *p* < 0.001), as well as diabetics (*r* = − 0.505, *p* < 0.001). A similar correlation was seen for HOMA-IR (non-diabetics *r* = − 0.203, *p* < 0.001; prediabetics *r* = − 0.419, *p* < 0.001; diabetics *r* = −0.417, *p* = 0.002), whereas no statistically significant difference was seen for HbA1c (non-diabetics *r* = −0.054, *p* = 0.290; prediabetics *r* = −0.147, *p* = 0.062; diabetics *r* = −0.042, *p* = 0.669).

## Discussion

Overt type 2 diabetes is well recognized as an indication for bariatric surgery for persons with morbid obesity. LRYGB was in our study associated with a reduction in fasting insulin, HbA1c, and fasting glucose levels as well as improvement in insulin resistance, not only in the diabetic group but also for all patients irrespective of glucometabolic state at the time of surgery. In patients with type 2 diabetes, a marked reduction was seen in all outcome measures, despite a wide distribution of insulin levels. Furthermore, consistent with previous studies, complete or partial remission rate of diabetes was high, at least 2 years after surgery. Patients classified as having prediabetes according to the definition of the American Diabetes Association [26], showed significant improvement, 84% having HbA1c and fasting plasma glucose values in the normal range 2 years after surgery.

These effects have been described previously in smaller studies reporting improvement in various aspects of glucose metabolism [12, 15–18, 20, 21]. Although the mechanisms that lie behind these effects of bariatric surgery are far from being established, they appear to be mediated by a combination of factors both dependent and independent of weight loss, including improved gastrointestinal endocrine function (e.g., increased GLP-1 secretion) [15, 17, 19, 30]. A significant association was seen between weight loss and improvement in the metabolic outcomes in the present study. This finding concurs with current evidence supporting a clear association between massive weight loss after bariatric surgery and improved insulin sensitivity [31, 32]. Weight loss-independent factors are more complex and probably explain the improvement in glucose homeostasis shortly after bariatric surgery [17, 19]. An increase in insulin level is associated with weight-gain in patients with diabetes [33], and a fall in insulin level may be a contributing factor to weight loss after bariatric surgery [34]. Furthermore, bariatric surgery may improve pancreatic beta-cell function [18, 21, 30]. This effect appears to be the most important among non-diabetics and patients with recent onset of diabetes where beta-cell dysfunction is only limited [18]. In the present study, the effect on prediabetes was significant. Although this specific group of patients is at slightly higher risk for postoperative complications compared with patients with normal glucose tolerance [35], the results of the present study support the effect of bariatric surgery in the prevention of new onset of diabetes [36, 37]. Improvement of metabolic risk factors associated with prediabetes and prevention of the otherwise high risk of conversion of prediabetes into diabetes are of fundamental value for the individual patient [23, 36–38]. Furthermore, with estimated annual costs in the USA for prediabetes of $43.4 billion and as high as $13,240 per patient if the prediabetes progress into diabetes, bariatric surgery may help save health-care expenditure as well. With a risk of progression from prediabetes to overt diabetes higher than 10% during the first 1.5 years after diagnosis [38], prediabetes should thus be recognized as a factor increasing the indication for bariatric surgery in patients with morbid obesity.

The metabolic benefits in patients with established type 2 diabetes were quite convincing, with marked improvements in fasting insulin, glucose, and HbA1c levels as well as a reduction in insulin resistance according to HOMA-IR. Furthermore, 62% of patients with diabetes at baseline were in complete or partial remission without medical treatment at the 2-year follow-up, and an additional 17% were in a well-controlled situation with HbA1c levels < 6.5%. These improvements are in line with the results of previous randomized trials [9, 10, 39, 40], as well as other large non-randomized trials [11, 32, 41].

### Strengths and Limitations

The study has several limitations that must be acknowledged. First, this is a retrospective study, which limits our analyses to patient-specific information and laboratory tests routinely collected at our department. Most importantly, oral glucose tolerance tests were not performed, limiting analyses to fasting samples and thus ruling out evaluation of the insulin response to a meal. The glucose tolerance test, however, is resource demanding and would have dramatically limited the number of participants in this study. Instead, we estimated insulin resistance by HOMA-IR. The euglycemic hyperinsulinemic clamp (EHC) is considered the gold standard for evaluation of insulin response. However, EHC is impractical in larger study cohorts due to the resources required. As a result, HOMA-IR is often used in larger epidemiologic studies, thus enabling comparison between studies. The use of HOMA-IR limits the interpretation of insulin resistance to the relationship between insulin and glucose at basal concentrations [23] but has been shown to correlate well with the EHC [42]. Uniform definitions of diabetes, prediabetes, and non-diabetes based on HbA1c values were used throughout the study. HbA1c reflects glucose levels during non-fasting hours of the day and is now accepted as a diagnostic criterion for type 2 diabetes mellitus [43]. The present study included over 500 participants with varying degree of glucose tolerance, with complete data both at baseline and 2 years after surgery, and this, to our knowledge, is the largest study to date on improvement in glucose metabolism after laparoscopic bariatric surgery. The study included only LRYGB procedures. Although similar effects on glucose homeostasis and incretin levels have been reported with LRYGB and sleeve gastrectomy [44], recent studies imply better glycemic improvement with LRYGB compared with sleeve gastrectomy for patients with type 2 diabetes [45]. The results of the present study can thus not be generalized to sleeve gastrectomy, and further comparisons for patients with prediabetes are needed.

## Conclusion

Insulin homeostasis and glucometabolic control improve in all patients after LRYGB, not only in diabetics but also in prediabetics and non-diabetic obese patients, and this improvement is sustained 2 years after surgery.

## References

[1] Hofso D, Jenssen T, Hager H, Roislien J, Hjelmesaeth J (2010). Fasting plasma glucose in the screening for type 2 diabetes in morbidly obese subjects. Obes Surg.

[2] Flum DR, Belle SH, Longitudinal Assessment of Bariatric Surgery C (2009). Perioperative safety in the longitudinal assessment of bariatric surgery. N Engl J Med.

[3] Mostaedi R, Lackey DE, Adams SH, Dada SA, Hoda ZA, Ali MR (2014). Prevalence of undiagnosed and inadequately treated type 2 diabetes mellitus, hypertension, and dyslipidemia in morbidly obese patients who present for bariatric surgery. Obes Surg.

[4] Stratton IM, Adler AI, Neil HA, Matthews DR, Manley SE, Cull CA, Hadden D, Turner RC, Holman RR (2000). Association of glycaemia with macrovascular and microvascular complications of type 2 diabetes (UKPDS 35): prospective observational study. BMJ..

[5] Tancredi M, Rosengren A, Svensson AM, Kosiborod M, Pivodic A, Gudbjörnsdottir S, Wedel H, Clements M, Dahlqvist S, Lind M (2015). Excess mortality among persons with type 2 diabetes. N Engl J Med.

[6] Nathan DM (2014). The diabetes control and complications trial/epidemiology of diabetes interventions and complications study at 30 years: overview. Diabetes Care.

[7] Jans A, Naslund I, Ottosson J, Szabo E, Naslund E, Stenberg E (2019). Duration of type 2 diabetes and remission rates after bariatric surgery in Sweden 2007-2015: a registry-based cohort study. PLoS Med.

[8] Stenberg E, Szabo E, Agren G, Näslund E, Boman L, Bylund A, Hedenbro J, Laurenius A, Lundegårdh G, Lönroth H, Möller P, Sundbom M, Ottosson J, Näslund I, Scandinavian Obesity Surgery Registry Study Group (2014). Early complications after laparoscopic gastric bypass surgery: results from the Scandinavian obesity surgery registry. Ann Surg.

[9] Schauer PR, Bhatt DL, Kirwan JP, Wolski K, Aminian A, Brethauer SA, Navaneethan SD, Singh RP, Pothier CE, Nissen SE, Kashyap SR, STAMPEDE Investigators (2017). Bariatric surgery versus intensive medical therapy for diabetes - 5-year outcomes. N Engl J Med.

[10] Mingrone G, Panunzi S, De Gaetano A (2012). Bariatric surgery versus conventional medical therapy for type 2 diabetes. N Engl J Med.

[11] Sjostrom L, Peltonen M, Jacobson P (2014). Association of bariatric surgery with long-term remission of type 2 diabetes and with microvascular and macrovascular complications. JAMA..

[12] Eickhoff H, Guimaraes A, Louro TM, Seica RM, Castro ESF (2015). Insulin resistance and beta cell function before and after sleeve gastrectomy in obese patients with impaired fasting glucose or type 2 diabetes. Surg Endosc.

[13] O'Brien R, Johnson E, Haneuse S, Coleman KJ, O'Connor PJ, Fisher DP, Sidney S, Bogart A, Theis MK, Anau J, Schroeder EB, Arterburn D (2018). Microvascular outcomes in patients with diabetes after bariatric surgery versus usual care: a matched cohort study. Ann Intern Med.

[14] Chen Y, Corsino L, Shantavasinkul PC, Grant J, Portenier D, Ding L, Torquati A (2016). Gastric bypass surgery leads to long-term remission or improvement of type 2 diabetes and significant decrease of microvascular and macrovascular complications. Ann Surg.

[15] Liaskos C, Koliaki C, Alexiadou K, Argyrakopoulou G, Tentolouris N, Diamantis T, Alexandrou A, Katsilambros N, Kokkinos A (2018). Roux-en-Y gastric bypass is more effective than sleeve Gastrectomy in improving postprandial glycaemia and lipaemia in non-diabetic morbidly obese patients: a short-term follow-up analysis. Obes Surg.

[16] Casella G, Soricelli E, Castagneto-Gissey L, Redler A, Basso N, Mingrone G (2016). Changes in insulin sensitivity and secretion after sleeve gastrectomy. Br J Surg.

[17] Campos GM, Rabl C, Havel PJ, Rao M, Schwarz JM, Schambelan M, Mulligan K (2014). Changes in post-prandial glucose and pancreatic hormones, and steady-state insulin and free fatty acids after gastric bypass surgery. Surg Obes Relat Dis.

[18] Purnell JQ, Johnson GS, Wahed AS, Dalla Man C, Piccinini F, Cobelli C, Prigeon RL, Goodpaster BH, Kelley DE, Staten MA, Foster-Schubert KE, Cummings DE, Flum DR, Courcoulas AP, Havel PJ, Wolfe BM (2018). Prospective evaluation of insulin and incretin dynamics in obese adults with and without diabetes for 2 years after Roux-en-Y gastric bypass. Diabetologia..

[19] Wallenius V, Dirinck E, Fandriks L, Maleckas A, le Roux CW, Thorell A (2018). Glycemic control after sleeve gastrectomy and Roux-en-Y gastric bypass in obese subjects with type 2 diabetes mellitus. Obes Surg.

[20] Camastra S, Muscelli E, Gastaldelli A, Holst JJ, Astiarraga B, Baldi S, Nannipieri M, Ciociaro D, Anselmino M, Mari A, Ferrannini E (2013). Long-term effects of bariatric surgery on meal disposal and beta-cell function in diabetic and nondiabetic patients. Diabetes..

[21] Bradley D, Conte C, Mittendorfer B, Eagon JC, Varela JE, Fabbrini E, Gastaldelli A, Chambers KT, Su X, Okunade A, Patterson BW, Klein S (2012). Gastric bypass and banding equally improve insulin sensitivity and beta cell function. J Clin Invest.

[22] Mazidi M, Gao HK, Li L, Hui H, Zhang Y (2017). Effects of Roux-en-Y gastric bypass on insulin secretion and sensitivity, glucose homeostasis, and diabetic control: a prospective cohort study in Chinese patients. Surgery..

[23] Andersson DP, Dahlman I, Eriksson Hogling D, Bäckdahl J, Toft E, Qvisth V, Näslund E, Thorell A, Rydén M, Arner P (2019). Improved metabolism and body composition beyond normal levels following gastric bypass surgery: a longitudinal study. J Intern Med.

[24] Olbers T, Lonroth H, Fagevik-Olsen M, Lundell L (2003). Laparoscopic gastric bypass: development of technique, respiratory function, and long-term outcome. Obes Surg.

[25] Stenberg E, Szabo E, Agren G (2016). Closure of mesenteric defects in laparoscopic gastric bypass: a multicentre, randomised, parallel, open-label trial. Lancet..

[26] American Diabetes A (2014). Diagnosis and classification of diabetes mellitus. Diabetes Care.

[27] Geistanger A, Arends S, Berding C, Hoshino T, Jeppsson JO, Little R, Siebelder C, Weykamp C, the IFCC Working Group on Standardization of Hemoglobin A1c (2008). Statistical methods for monitoring the relationship between the IFCC reference measurement procedure for hemoglobin A1c and the designated comparison methods in the United States, Japan, and Sweden. Clin Chem.

[28] Matthews DR, Hosker JP, Rudenski AS, Naylor BA, Treacher DF, Turner RC (1985). Homeostasis model assessment: insulin resistance and beta-cell function from fasting plasma glucose and insulin concentrations in man. Diabetologia..

[29] Brethauer SA, Kim J, El Chaar M (2015). Standardized outcomes reporting in metabolic and bariatric surgery. Surg Obes Relat Dis.

[30] Chondronikola M, Harris LL, Klein S (2016). Bariatric surgery and type 2 diabetes: are there weight loss-independent therapeutic effects of upper gastrointestinal bypass?. J Intern Med.

[31] Mingrone G, Cummings DE (2016). Changes of insulin sensitivity and secretion after bariatric/metabolic surgery. Surg Obes Relat Dis.

[32] Sjoholm K, Sjostrom E, Carlsson LM, Peltonen M (2016). Weight change-adjusted effects of gastric bypass surgery on glucose metabolism: 2- and 10-year results from the Swedish obese subjects (SOS) study. Diabetes Care.

[33] Carlson MG, Campbell PJ (1993). Intensive insulin therapy and weight gain in IDDM. Diabetes..

[34] Ludwig DS, Ebbeling CB (2018). The carbohydrate-insulin model of obesity: beyond “calories in, calories out”. JAMA Intern Med.

[35] Stenberg E, Szabo E, Naslund I (2014). Scandinavian obesity surgery registry study G. is glycosylated hemoglobin A1 c associated with increased risk for severe early postoperative complications in nondiabetics after laparoscopic gastric bypass?. Surg Obes Relat Dis.

[36] Carlsson LM, Peltonen M, Ahlin S (2012). Bariatric surgery and prevention of type 2 diabetes in Swedish obese subjects. N Engl J Med.

[37] Backman O, Bruze G, Naslund I (2019). Gastric bypass surgery reduces de novo cases of type 2 diabetes to population levels: a nationwide cohort study from Sweden. Ann Surg.

[38] Rasmussen SS, Glumer C, Sandbaek A, Lauritzen T, Borch-Johnsen K (2008). Determinants of progression from impaired fasting glucose and impaired glucose tolerance to diabetes in a high-risk screened population: 3 year follow-up in the ADDITION study, Denmark. Diabetologia.

[39] Ikramuddin S, Korner J, Lee WJ, Connett JE, Inabnet WB, Billington CJ, Thomas AJ, Leslie DB, Chong K, Jeffery RW, Ahmed L, Vella A, Chuang LM, Bessler M, Sarr MG, Swain JM, Laqua P, Jensen MD, Bantle JP (2013). Roux-en-Y gastric bypass vs intensive medical management for the control of type 2 diabetes, hypertension, and hyperlipidemia: the diabetes surgery study randomized clinical trial. JAMA..

[40] Dixon JB, O'Brien PE, Playfair J, Chapman L, Schachter LM, Skinner S, Proietto J, Bailey M, Anderson M (2008). Adjustable gastric banding and conventional therapy for type 2 diabetes: a randomized controlled trial. JAMA..

[41] Adams TD, Davidson LE, Litwin SE, Kolotkin RL, LaMonte MJ, Pendleton RC, Strong MB, Vinik R, Wanner NA, Hopkins PN, Gress RE, Walker JM, Cloward TV, Nuttall RT, Hammoud A, Greenwood JLJ, Crosby RD, McKinlay R, Simper SC, Smith SC, Hunt SC (2012). Health benefits of gastric bypass surgery after 6 years. JAMA..

[42] Tosi F, Bonora E, Moghetti P (2017). Insulin resistance in a large cohort of women with polycystic ovary syndrome: a comparison between euglycaemic-hyperinsulinaemic clamp and surrogate indexes. Hum Reprod.

[43] World Health Organization. In: Use of Glycated Haemoglobin (HbA1c) in the Diagnosis of Diabetes Mellitus: Abbreviated Report of a WHO Consultation, Geneva; 2011. 26158184

[44] Peterli R, Wolnerhanssen B, Peters T (2009). Improvement in glucose metabolism after bariatric surgery: comparison of laparoscopic Roux-en-Y gastric bypass and laparoscopic sleeve gastrectomy: a prospective randomized trial. Ann Surg.

[45] Hofso D, Fatima F, Borgeraas H (2019). Gastric bypass versus sleeve gastrectomy in patients with type 2 diabetes (Oseberg): a single-Centre, triple-blind, randomised controlled trial. Lancet Diabetes Endocrinol.

